# Evaluating Processing Parameter Effects on Polymer Grades and Plastic Coloring: Insights from Experimental Design and Characterization Studies

**DOI:** 10.3390/polym16233409

**Published:** 2024-12-03

**Authors:** Jamal Alsadi, Ameen Alawneh, Ahmed Ali Khatatbeh, Mutaz Abdel Wahed, Mustafa Alseafan, Saleh Alomari

**Affiliations:** 1 Faculty of Engineering, Jadara University, Irbid 21110, Jordan; seafan@jadara.edu.jo; 2 Faculty of Science & Information Technology, Jadara University, Irbid 21110, Jordan; ameen.al@jadara.edu.jo (A.A.); mutaz@jadara.edu.jo (M.A.W.); omari08@jadara.edu.jo (S.A.); 3 Faculty of Engineering, Al-al Bayt University, Mafraq 25113, Jordan; khatatbeh@aabu.edu.jo

**Keywords:** polymer, additives, color, DoE, rheology, SEM, FTIR characterization

## Abstract

Resolving material and processing issues is essential to improving formulations and attaining accurate color matching in blending. Two transparent polycarbonate resins made up the blend: PC1, which made up 33% of the mixture with a Melt Flow Index (MFI) of 25 g/min, and PC2, which made up 67% of the mixture with an MFI of 65 g/min. This study employed data mining and a well-structured experiment using Design of Experiment (DoE) software-8 to investigate the effects of various processing temperatures on identical material formulations. The primary objective was to understand the influence of the operating conditions on the viscosity of a red letdown pigment made of polycarbonate, both with additive (WA) and without additive (WOA). The study analyzed the effects of processing parameters and rheological properties using a co-rotating twin-screw extruder. Rheology, Fourier-Transform Infrared Spectroscopy (FTIR), and Scanning Electron Microscopy (SEM) were employed to compare results and evaluate their impact on minimizing color discrepancies and improving Single-Pass Color Uniformity.

## 1. Introduction

Plastics manufacturing has developed significantly, driven by the adaptability and cost-effectiveness of plastics like polycarbonate (PC), known for its transparency, strength, and heat resistance. Nevertheless, accomplishing steady color matching in PC manufacturing remains in demand due to factors like coloring formulation and environmental impacts. This study addresses these challenges by examining the effects of various processing conditions on PC blends with additives and composites, focusing on color consistency. Using methods such as color testing, blending, extrusion, rheology, FTIR, and SEM, along with statistical tools like DOE and RSM, this research optimizes processing parameters to enhance color accuracy and quality in colored polymer products [1,2,3,4].

Current progress in blending pigment with polymer composites has highlighted the importance of optimizing distribution and dispersion techniques, enhancing functioning through additives, and refining processing conditions employing statistical procedures. This focus objects to achieving precise color uniformity and stability in processing plastic, addressing both artistic and efficient necessities. With the rising importance of sustainability, the investigation into bio-based dyes for incorporation with traditional polymers is increasing, preparing the way for advanced solutions that develop color consistency and execution [1,2,3,4].

Temperature parameters are vital variables that can influence these letdown proportions. Factors such as viscosity variations, color improvement, melt flow behavior, thermal stability, and energy input produce a complex relationship between temperature parameters and pigment distribution in plastic composites. Reducing waste and expediting material delivery are the largest challenges facing polymer compounders, especially individuals who provide small lots of materials with rapid lead times for businesses working on the development of prototypes [5]. Compounding is the process of homogeneously mixing pigments in predetermined amounts and ratios to create the desired color. These pigments, typically different colors, reflect the incident light at various depths, resulting in the desired color being seen as the result of the combined effect of reflection. However, variations within the source of light, the observer, the reflecting object, and other factors can all impact how a color is perceived. Color labeling identifies colors to classify elements, increasing identification and following in the plastics industry [6,7]. It confirms reliable pigment usage, decreases errors, and improves quality control by distinguishing additives, pigments, and polymers during sample preparation. This approach also supports batch tracking, defect detection, and maintaining product standards, ultimately leading to higher customer satisfaction [6,7]. Color deviations can result from a variety of factors, such as differences in formulations or pigment dispersions and the effects of degradation behavior and processing parameters on material performance. The rheological properties, and thus the ability of pigments to disperse in the resin, are further enhanced by the use of widely applied processing aids [8,9].

This is a unique opportunity to examine the results of these variations in color combinations when compounding. According to the CIE L*, a*, and b* color values obtained using a spectrophotometer, we present the variations involved in such adjustments. The lab color-space components are L*, a*, and b*. The color-opponent space with L* represents lightness and a* and b* represent the color opponents on the green–red and blue–yellow axes, respectively [8,9].

It takes the proper amount of energy to reach optimal dispersion. Even though there are many tools available for this, a straightforward rise in temperature can help to quicken the intermolecular interactions among the system’s components. It is essential that the pigment is not vulnerable to higher processing temperatures for a long period, in spite of the fact that colorant constancy fluctuates depending on the kind of polymer. Accordingly, it is essential to recognize the properties of both the pigment and the polymer beforehand. By using this knowledge, degradation brought on by high processing temperatures or exposure to the environment can be avoided [10].

According to historical data, it is occasionally necessary to make several adjustments to the processing parameters before the desired color can be successfully obtained. 

By conducting an efficient analysis of pigments, additives, and resins and examining how various interactions and processing conditions influence these, this study aims to uncover the underlying science [11].

Moreover, the incorporation of letdown ratios of pigments during plastic compounding is subject to the influence of processing temperature and refers to the proportion of pigment or additive to the base polymer in a compound, playing a pivotal role in achieving the desired color or performance characteristics in the final plastic product such as by reducing the amount of pigment loading in the concentrate using additives and plasticizers to make the resin more lubricant and lower the viscosity of the melt and reducing the molecular weight grade of the polymer with an increased melt flow compared to the letdown of the polymer [12].

The incorporation of additives into polymeric components has potentially introduced unexpected impacts on their viscosity, mechanical properties, and aesthetic appearance [13,14]. Numerous researchers have investigated the addition of color to pristine resin. Rheological studies are commonly employed to analyze polymers, blends, and various semi-solid, solid, and fluid materials. Moreover, rheological properties play a crucial role in bridging the different processing stages to achieve the final state of the product [15,16]. The experimental design utilized the full factorial Response-Surface Method (RSM), incorporating three stages of process variables and a range of processing conditions denoted as General Trends (GTs). Both RSM and GTs effectively generated process parameters in a statistically significant manner, as documented in previous studies [17,18,19]. This study specifically emphasizes how, within the defined range of processing conditions, an optimized combination of temperature, feed rate, and speed values showed a slight deviance from the expected color output. Furthermore, more research is required to determine the best processing parameters for various color formulations and grades, which will significantly reduce waste, as shown by previous research [17,18,19].

Nowadays, numerous academic researchers have concentrated on evaluating the processing conditions of polymer grades and plastic coloring’s properties, drawing insights from comprehensive experimental designs and characterization works. Furthermore, recent research has examined the impact of varying processing parameters on insert-injection molded polypropylene (PP) single-polymer composite (SPC) parts, highlighting barrel temperature as the most influential factor, followed by holding time and injection pressure. Another study focused on developing PC-based extrusion materials for MEX by blending PC with poly (butylene adipate-co-terephthalate) (PBAT), significantly enhancing PC’s processing capabilities by up to 250 °C. Additionally, studies emphasized the critical role of injection molding machine (IMM) performance in manufacturing, noting that despite data collection advancements, comprehensive analyses of machine-specific behaviors remain limited [20,21,22].

Several recent studies have examined the rheological characteristics of PC/ABS blends across different ratios and temperatures. Noting enhanced heterogeneity in ABS-rich blends with increased temperature and reduced heterogeneity in PC-rich blends. They also compared the rheological and mechanical properties of fossil-based and bio-based PC, finding distinct behaviors in viscosity, storage and loss moduli, and mechanical performance. An additional study investigated rheology’s effect on the process of extrusion and injection molding, highlighting structural optimization for developed material quality performance in polymer processing. Furthermore, a detailed analysis highlighted current developments in polymer processing, rheology, and composites, encompassing polymer science, biopolymers, and simulation, and the advancement of these fields [23,24,25,26].

The grade without additives and pigment (WOP) exhibits higher viscosity than the process with the TSE (Coperion, Stuttgart, Germany). Conversely, the grade blends with additives and pigment (WP) result in the lowest viscosity; this difference is attributed to the additives [27]. Numerous researchers have examined the degradation pathways of polymers. Photo-oxidative degradation of weathered plastic constituents results from the combined effects of UV light (295 nm to 400 nm), moisture, and heat, all present in solar spectra [28]. Ongoing research continues to investigate the underlying chemistry. In outdoor applications where bisphenol-based polycarbonate (PC) is used, exposure to oxygen, sunlight, and humidity can lead to polymer degradation, resulting in mechanical failure and the loss of optical and aesthetic properties [4,29,30]. Moreover, UV aging led to a decline in the material’s performance under uniaxial tension. This reduction in PC’s mechanical properties was linked to the rupture of its most vulnerable bonds, as shown by FTIR analysis, and to surface cracks visible in OM and SEM micrographs [31,32].

Investigating complex relationships between variables is another benefit of RSM, which enhances material performance and produces fresh insights [33]. Recently, many researchers have successfully improved performance characteristics in polymer materials by using RSM to statistically model and optimize polycarbonate composites’ properties [34].

The application of RSM has proven effective in improving polycarbonate nanocomposites’ performance by analyzing the interactions among influential factors affecting material properties [35].

Many researchers have previously investigated the processing parameters’ effects on properties like salt rejection and water permeability, with findings validated through the analysis of variance (ANOVA). Models created through DoE emphasize the strong influence of these parameters on material properties, aiding in the optimization of polycarbonate and pigment blending for improved performance. The DoE approach was used to develop additional models. Response-Surface Methodology (RSM) was effectively employed to optimize the preparation conditions for the multifunctional polyacrylic acid–graphene oxide composite. These models were then validated through analysis of variance (ANOVA) [33,34,35,36].

This research focuses on blending two polycarbonate resins to achieve consistent color properties in plastics. It evaluates the impact of processing parameters such as temperature, speed, and feed rate on pigment dispersion, using Design of Experiments (DoE) and Response-Surface Methodology (RSM) for optimization. By integrating historical industrial data, it addresses challenges in color consistency, contributing to improved manufacturing of colored polymer products [37,38,39].

The primary objective is to explore how processing parameters such as speed, temperature, and feed rate affect consistent color output in polycarbonate materials [40]. Research highlights that the final structure of MWNT/glass fiber composites is significantly affected by the initial dispersion of multi-walled carbon nanotubes (MWNTs) and the structure of the porous media. These factors directly influence the composite’s homogeneity and overall material properties, emphasizing the importance of controlled dispersion techniques for achieving desired mechanical and thermal characteristics in the composite [40]. An additional study demonstrated that adding low-density polyethylene (LDPE) to polycarbonate (PC) enhances injection molding processability by creating interfacial slippage, which reduces overall viscosity [40].

Several studies on pigment brand, content, and processing parameters demonstrate their influence on color uniformity and product quality, highlighting that actual color strategies can be critical for success in customer shops. This study intersects with comprehensive debates on color psychology, emphasizing how color touches purchaser perception and choice, which is extremely related to marketing and design perceptions. Additional investigation into color psychology can offer insights for enhancing color plans in product improvement [41].

Finally, the study explores how processing temperature affects pigment dispersion and letdown ratios in polymer matrices, addressing factors such as viscosity and thermal stability [42,43]. The researchers investigated the rheological behavior and processability of multiwalled carbon nanotubes (MWNTs) in epoxy by preparing suspensions with different dispersion qualities, aspect ratios, concentrations, network structures, and orientations. They measured the rheological properties using a cone and plate rheometer to analyze the effects of these variables on the MWNT–epoxy suspensions [44]. Recent research on polycarbonate (PC)/multiwalled carbon nanotubes (MWNT) composites’ rheological characterization by frequency sweeps at 280 °C were performed using a master batch dilution technique [45,46].

Scanning Electron Microscopy (SEM) provides valuable insights into the microstructural features of polycarbonate blends, particularly for analyzing pigment dispersion and agglomeration. The findings indicate that polycarbonate (PC) behaves in a mostly Newtonian manner, while acrylonitrile butadiene styrene (ABS) demonstrates significant shear thinning [47,48].

This work presents a technique for precisely utilizing SEM to analyze particle size, distribution, and surface morphology in high-viscosity polymer emulsions. The technique resolves challenges in SEM analysis caused by high viscosity, allowing clear observation of emulsion micromorphology across various viscosities [48,49].

Furthermore, Scanning Electron Microscopy (SEM) is utilized to demonstrate the relationship between pigment dispersion quality and color consistency, showing that uniform dispersion helps to prevent agglomeration and color discrepancies [49].

Whereas some academic scientists have investigated the relationship between color deviation and color dispersion [50,51], the complete exploration of the effects of varying processing parameters, particularly regarding polycarbonates (PC), still needs to be discovered. Therefore, the study’s objective is to determine how adaptations in processing parameters affect the chosen color output.

Consequently, this work aims to examine the relationships between processing parameters, pigment dispersion, and color consistency in compounded plastic materials, particularly within polycarbonate matrices. We will employ Design of Experiments (DOE) and Response-Surface Methodology (RSM) to systematically optimize pigment blending processes, enhancing our understanding of how processing conditions influence color stability and consistency. The research will focus on optimizing the blending of two polycarbonate (PC) resins with distinct melt flow indices (MFIs) of 25 kg/min and 6.5 kg/min. To achieve this, we will conduct 15 experimental runs, systematically varying the extrusion temperature (230 °C, 255 °C, and 280 °C), speed (700 rpm, 750 rpm, and 800 rpm), and feed rate (20 kg/h, 25 kg/h, and 30 kg/h).

To assess processing parameters’ impact on color consistency and the dispersion effects on the physical and chemical structures of the materials, we will utilize FTIR and SEM. Additionally, we will examine the influence of processing temperatures on viscosity and color matching in the polycarbonate (PC) blends, focusing on the rheological properties of the blends with and without additives. Using Design-Expert software-8, we will optimize the processing conditions to minimize color variations and enhance first-pass color accuracy. The goal is to enhance the quality and uniformity of color formulations based on polycarbonate by establishing a correlation between color outputs and viscosity data. This study offer deeper insights into blending processes; hopefully, this lays the groundwork for future studies in polymer composites.

## 2. Materials and Methods 

This section outlines the materials and equipment used in the experiments and discusses the procedures followed. The aim is to understand the formulation of the letdown pigment in the master batch, the characterization equipment used, and how processing parameters affect a red letdown pigment created from a master batch with 33% PC1 and 67% PC2, using a pigment ratio of 0.01:1. The formulation involves blending the colorant with the base resin in various ratios, typically up to 1:0.01 for pure resin master batches. The design of the master batch depends on the processing parameters, pigment loading, and the desired color in the final plastic product. This section details the materials, sample preparations, and methods used in the experiments, including the procedures for preparing the master batch and determining the letdown ratio (LDR).

### 2.1. Letdown Ratio (LDR) in Master Batch

The letdown pigment P1L01 comprises 99 parts resin and 1 part red pigment. P1L01 helps to disperse pigments in polycarbonate (PC) mixtures, mixing small pigment amounts and simplifying the weighing. By this technique, accurate color formulation is guaranteed, particularly for minute pigment quantities. The bulk use of pigment would render the plastic opaque, while combining 1% pigment with 99% resin yields a transparent chip. Proper dispersion requires melting concentrates at or below the compounding temperature, matching the pure resin’s viscosity. Higher pigment levels increase viscosity, potentially leading to polymer breakdown and frictional heat.

To study the impact of processing parameters on red letdown pigment in PC, ratios are formulated by blending materials in specific proportions, up to 1:0.01 for a master batch. The master batch design is based on the processing parameters, pigment loading, and desired final color. For a master batch with 33% PC1, 67% PC2, and a 1:0.01 pigment ratio, we prepare 1010 g total by mixing 330 g of PC1, 670 g of PC2, and 10 g of pigment. We pre-mix the pigment with some PC, then blend with the rest. We process through an extruder, cool, and pelletize. For a 1% pigment concentration, we mix 1 part master batch with 100 parts base polymer. We test and adjust samples for color and dispersion to achieve the desired final product. 

### 2.2. Extrusion Processing of Grade 3

This material achieves vibrant colors due to its composition, which combines PC1 and PC2—two translucent polycarbonate resins. Resin 1, constituting 33% of the blend, has a 25 g/10 min Melt Flow Index (MFI), while Resin 2, which makes up 67% of the blend, has a 6.5 g/10 min MFI. Table 1 details the incorporation of five pigments and various additives into both resins. The materials were extruded using a 25.5 mm Coperion twin co-rotating screw extruder. Color additives, comprising pigments and additives, constituted 1% of the total weight. The additives and pigments were blended with the resins at a ratio of 100:01 using a super floater. The compounding process utilized a Coperion twin-screw extruder from Germany with nine heating zones for the barrel and one for the die. 

The experimentation involved utilizing a Grade 3 co-rotating twin-screw extruder with a power rating of 27 kW (ZSK26 of Coperion Germany). There were 10 heating zones present in the extruder: nine indicated on the barrel and one at the die. The desired color output was ascertained in terms of CIE L*, a*, and b* values using a spectrophotometer (Macbeth 7000A (Standard Observer Function), D65 light, and 1964-10). In terms of CIE L*, a*, and b* values, the desired color output is L* = 68.5, a* = 1.43, and b* = 15.69. 

The available historical records determined the resin and pigment selection. The rpm, the feed rate to the extruder, and the heating zone temperature were the three process parameters that were taken into account. The parameters were varied at five different levels. It is important to keep in mind that these grades were generated in the middle, or level three, of operating conditions. After extrusion, plastic strands were cooled in water, dried, and pelletized. Each production batch’s pellets were injection-molded into three sample chips for color coordinate analysis using the Color-Eye^®^ 7000A spectrophotometer from X-Rite (Grand Rapids, MI, USA). After molding, specimens were dried at room temperature for optical microscopy tests, as well as further characterization. 

### 2.3. Color Chip Samples

These pellets were molded via injection molding into three rectangular color chips measuring 3 × 2 × 0.1 inches. The injection-molding machine was a Kawaguchi Co. KM100 (Fujimatsu City, Aichi Prefecture, Japan) model with 85 tons of clamp tonnage, comprising injection and clamping components. Samples were produced for subsequent characterization and color quantification, processed at around 28 MPa (1000 PSI) and 280 °C. The specimens were later air-dried at room temperature in the laboratory to facilitate optical microscopic tests, spectrometer color readings, and characterization measurements using a spectrometer, rheometer, and FTIR.

### 2.4. SEM Sample Preparation

Properties were quantified using SEM. Microscopic sample preparations were performed using a microtome. For other samples, a fully automatic Slee rotary microtome (CUT 6062) was used to cut thin slices, yielding slices as thin as 5 microns. Slices were cut into 50-micron-thick chips for optical microscope scanning tests using SEM.

### 2.5. Experimental Testing Equipment 

Various equipment was employed for compound processing and sample preparation, rheological characterization, color quantification, and microstructural characterization.

#### 2.5.1. Mixer

A 3D material movement super floater (model SFC-50) manufactured by KAWATA MFG CO. Ltd. (Osaka, Japan) was used for dry blending. This mixer produces a high degree of mixing for various materials with differing densities and was used to prepare compounds before melting compounding in the extruders.

#### 2.5.2. Twin-Screw Extruder 

Compounding was carried out using a twin-screw extruder (TSE) manufactured by Coperion, Germany. This intermeshing, co-rotating twin-screw extruder had a 25.5 mm screw diameter, an L/D ratio of 37, and a 27 kW motor. It featured nine heating zones for the barrel and one for the die. The extrudate was quenched in cold water upon exiting the die, dried using air, and then converted into pellets via a pelletizer. The study utilized a Coperion intermeshing co-rotating twin-screw extruder (TSE) with a 25.5 mm screw diameter, featuring nine heating zones for precise temperature control. Post-extrusion, the material was quenched, dried, and pelletized before being injection-molded into color chips with a Kawaguchi KM100 machine operating at 280 °C and 28 MPa. Key processing parameters included a temperature range of 230–280 °C, feed rates of 20–30 kg/min, and variable screw speeds from 700 to 800 rpm, ensuring effective mixing and material homogeneity throughout the process.

#### 2.5.3. Rotational Rheometer

TA Instruments’ Ares-G2 rheometer was utilized. It has a transducer of its own. It is an example of technology that makes pure rheological measurements possible. The Forced Convection Oven (FCO) can be mounted on the test station’s side.

We used parallel plate geometry. We perform the dynamic viscosities’ measurements in a parallel-plate fixture (a diameter of 25 mm and a 1.0 mm gap size). The study sheared the testing sample between the two plates. The viscosity measurement was the result of the ratio of the deformation rate and the applied stress. This rheometer has specialized software to record data, program measurements, and show results. This rheometer offers an extensive range of new features, including excellent accuracy concerning data, fast data sampling, new TRIOS software Version 5.1, and a new large amplitude oscillatory shear (LAOS) test.

#### 2.5.4. FTIR—Perkin Elmer Spectrum 

FTIR microscopy is highlighted for its versatility in analyzing complex, multicomponent, and multilayer samples, especially in heritage materials. While FTIR spectroscopy provides non-destructive insights into layered structures, it lacks detailed chemical characterization of each layer. Developing FTIR microscopy-based methods for artwork analysis promises to complement these capabilities by offering precise chemical information on ancient artists’ materials. This approach guides experimental design for optimal color outcomes because it facilitates comparative assessments with rheological characteristics.

Functional groups and characteristic absorption results were identified for compounded plastic. This resulted in the PC grade batches without pigment and additives (WOA), and the combination of pigments and additives (WA) of the two polycarbonate resins used in FTIR characterizations (%PC1/%PC2) was 33%/67%. The chemical structures of the plastic composites were analyzed using an FTIR-Perkin Elmer spectrum 100-spectrometer, which recorded between 400 and 4000 cm^−1^ in the wave number range.

#### 2.5.5. Microscopy Examinations (SEM)

Microscopy examinations were carried out utilizing a Scanning Electron Microscope (SEM), Model Joel 5500 LV, to observe the microstructures of the produced compounds compared to the measurement of the color chips and to characterize SEM and FTIR readings.

#### 2.5.6. Spectrophotometer

A spectrophotometer is an instrument used for photometric measurements, which can assess spectral transmittance, spectral reflectance, or relative spectral emittance. Color measurements were conducted using a Spectrophotometer CE-7000A, which utilized X-Rite Color^®^ Master software Version 7.2.1. The Standard Observer Function employed was 1964–10° with D65 illumination. Measurements were taken at three different locations on each specimen (coupon) to derive tristimulus values (L*, a*, and b*). The target values for L*, a*, and b* were set at 68.5, 1.43, and 15.7, respectively. Color differences were subsequently calculated as dL*, da*, db*, and dE*.

### 2.6. Historical Data Mining

From the historical records of the year 2009, the initial data mining findings for the first nine months reveal the grades that experienced the most significant rate of adjustment when mixed with either diluted red pigment (P1L01) or red pigment (P1), as shown in Table 2. For example, the PC grade G3, which is translucent and contains diluted red pigment (P1L01), experienced the highest rate of adjustment.

**Figure 1:**
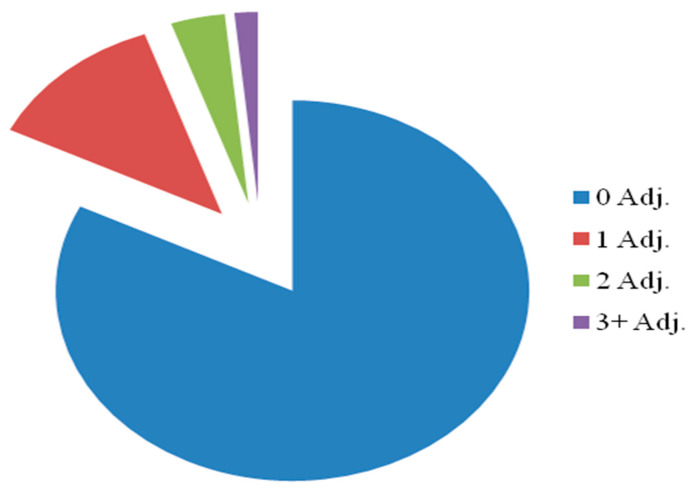
Illustrating the percentage of adjustment of the lots.

In the context of pigments, the letdown ratio refers to the ratio of the amount of pigment added to a mixture or formulation compared to the total amount of the mixture or formulation. It is often used in industries like paint manufacturing or printing to describe the proportion of pigment to the overall material. Seven out of the thirty-six (19.44%) modifications brought about by the pigment P1L01 involved Grade 3, as shown in Figure 1. Consequently, this grade has been employed for further experimental characterization and Design of Experiments (DoE) testing to analyze and reduce color mismatching in subsequent stages.

### 2.7. Methods 

#### 2.7.1. Processing Parameters Setup (General Trends) 

After extrusion, pellets were produced under three different parameters—temperature, speed, and feed rate—each varied at three levels (high, medium, and low) while keeping the other parameters constant. This experimental setup, known as General Trends (GT), involved temperatures of 230 °C, 255 °C, and 280 °C, with the speed and feed rate set at 750 rpm and 25 kg/h, respectively. Similarly, speed was tested at 700 rpm, 750 rpm, and 800 rpm, and feed rates at 20 kg/h, 25 kg/h, and 30 kg/h. Using DoE with these variables, this study aims to identify optimal process values for consistent color output in the plastic product.

#### 2.7.2. Response-Surface Methods (RSMs) 

Response-Surface Methodology (RSM) employs designs like full factorial, central composite (CCD), Box–Behnken, and D-optimal, optimizing processes with key factors. DoE identified variables for a process window model, determining the optimal experiment count. Evaluating the speed, temperature, and feed rate effects on output parameters, this study tested various grades to understand controlled parameter impacts on plastic color. The results aimed to optimize processing for efficiency in waste reduction and timely orders, suggesting a 3-level full factorial DoE for experimentation.

#### 2.7.3. Three-Level Factorial Design

The study examined the effects of temperature, feed rate, and screw speed (rpm) by implementing 15 distinct treatments. Each parameter was tested at three levels using a complete factorial design to analyze their impact on color outcomes. Table 3 illustrates the experimental design levels applied to the three process parameters: temperature, feed rate, and screw speed rpm. This study utilized a complete factorial experimental design incorporating three levels for each parameter to assess their impact on color outcomes. 

The specified color output, defined by CIE L*, a*, and b* values (L* = 68.5, a* = 1.43, and b* = 16.69), underwent analysis with Design-Expert^®^ Software (Version 8, Stat-Ease Inc., Minneapolis, MN, USA). Statistical methods, including ANOVA, were utilized to evaluate the significance of processing parameters and their interactions. Optimizing the processing parameters that impact color properties and developing a predictive equation for L*, a*, and b* values were the goals.

#### 2.7.4. Model Development

In order to produce consistent results, compounders seek to understand process variable–output color relationships fully. Design of Experiments (DoE) provides a systematic approach for planning experiments and establishing causal relationships, widely adopted across scientific disciplines for its ability to streamline experimental processes. Process optimization using the Response-Surface Method (RSM) employed a 3-level factorial design. The initial phase involved meticulously designing experiments to efficiently evaluate model parameters post-experimentation. Subsequently, a second-order polynomial mathematical model was developed to analyze the responses [52]. Regression analysis was conducted between the response and the independent variables to fit the empirical second-order polynomial model, as demonstrated by the following equation:

(1) $$y={\;\beta }_{o}+\sum_{i=1}^{k}{\;\beta }_{i}x_{i}+\sum_{i=1}^{k}\beta_{\mathrm{ii}}x_{i}^{2}+\sum_{i}\sum_{j>i}{\;\beta }_{\mathrm{ij}}x_{i}x_{j}+\;\varepsilon$$

where y is the predicted response; 

β_0_ is a constant term; β_i_ is the *i*th linear coefficient; 

β_ii_ is the *i*th quadratic coefficient; β*_ij_* is the *i*th interaction coefficient;

*x_i_* is the independent variable; k is a number of factors; and ε is the error. Any deviation beyond permissible limits between the target color and the actual output color can be identified as a color mismatch, measured using delta values such as dL, da, db, or dE, which quantify the Euclidean distance of color deviation in the 3D color space. RSM will produce statistical models for these responses and optimized variable values for maximizing individual responses. For that, the experiment was designed by composing a set of 15 experimental runs.

## 3. Results and Discussions

The primary objective of this research is to enhance color matching in polycarbonate (PC) grades, which involves examining the effects of incorrect formulations, poor pigment dispersion, processing conditions, and material interactions. Various scientific methodologies were developed and applied, including optimizations through data mining, processing parameters, Design of Experiments (DoE), and material formulations, as well as rheological and morphological characterizations. These methods were used to determine a compounded polycarbonate grade’s optimal processing parameters and color properties. This study investigated the impact of three processing parameters—temperature, speed, and feed rate—on the final product color.

### 3.1. Effect of Processing Parameters on Color and Design of Experiments (DoE)

For the highly adjusted grade, DoE was carried out to determine the material and processing parameters that cause the disparity in color—investigating correlations between the processing parameters (temperature, feed rate, and speed) and the color difference using one variable. The plotting of general trends and the execution of the experiments in accordance with the Design-Expert software-8 was conducted. Three processing conditions (temperature, feed rate, and speed) were used in the experimental design, resulting in 15 runs across different polycarbonate grades (see Table 4) for each variable while keeping the other two constant.

The results of this study show that variations in processing conditions significantly impact the desired color output. They also show that, depending on the operating conditions, grade–color combinations react in contrast to the intended color output. It also emphasizes how variations in processing parameters and tri-stimulus values like L*, a*, b*, and dE* interact. Above all, this analysis shows that there is an ideal range of temperature, feed rate, and rpm values that shows the least amount of variation from the intended color output within a given range of processing parameters. By conducting additional research, the finest processing condition combinations for different PC grades and color formulations can be found, resulting in a notable decrease in waste.

The majority of levels display a noteworthy response to changes in rpm, indicating high sensitivity to rpm increases. As rpm rises, there is a significant and abrupt decrease in dE* values. It is evident that elevating temperatures and increasing rpm rates yield almost identical effects on dE*. Once again, at level three, the dE* values indicate minimal deviation from the target color output, except possibly at rpm = 800 (level five), where degradation may occur; this phenomenon facilitates the dispersion of letdown pigment, polycarbonate resin, or additives (see Table 4).

For Grade 3, there is an observable shift in the dE* color-change pattern at different feed rates. However, at higher feed rates, like level five, a notable decrease was observed in dE* values. This phenomenon may be attributed to improved dispersion, as higher feed rates result in increased shear, enhancing the dispersion of letdown pigment through higher flow, as illustrated in the feed rate variation data in Table 4.

#### 3.1.1. Impact of Temperature Changes

**Figure 2:**
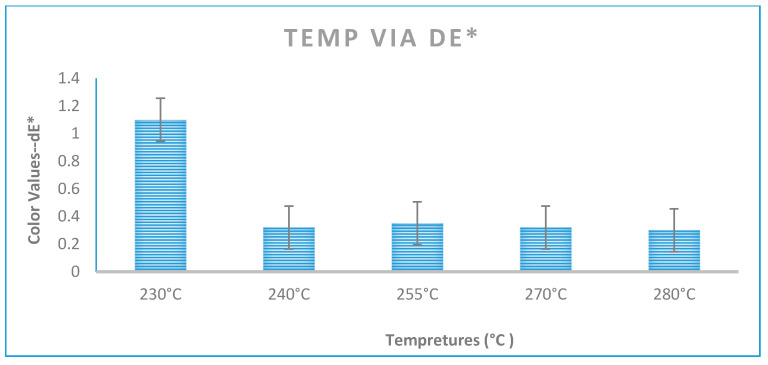
Result of processing temperature on color.

The variation in temperature has a noticeable impact on dE* values, as depicted in the temperature variation in Figure 2. At levels 2, 3, 4 and 5, the dE* values show a decreasing trend. This trend could be attributed to a decrease in viscosity resulting from a mixture of shear and elevation temperature in the formulation’s resin, pigments, or additives. Figure 2 illustrates the impact of changes in processing temperatures on the color output across PC Grade 3, represented in terms of CIE dE* values. 

The reduction in viscosity induced by higher shear enhances the pliability of the compounded material. Also, this reduction improves letdown pigments’ dispersion in the melting compound. As temperature increases, dE* values decrease significantly. However, this is not the case for level 1, where no improvement is observed. This exception is likely attributed to experimental variations and is not considered highly significant. Notably, level 5 displays the lowest dE* value. A significant reduction in waste is achieved by selecting an optimal set of processing conditions that minimize deviation from the intended color results, as exemplified by the temperature effect magnitude for Grade 3 at level five in Figure 2 and Table 4. 

#### 3.1.2. Regression Models Development

The present study focuses on using Response-Surface Methodology (RSM) to determine the optimal output color. The statistical combination of independent variables and the analysis of variance for the experimental results and their responses are presented in Table 4. This study investigated the impact of three processing conditions—temperature, feed rate, and screw speed (rpm)—at three levels (high, medium, and low) using a three-level factorial design. This experimental design was applied to different polycarbonate grades, varying one parameter at a time while keeping the other two constant. Each parameter was tested to analyze its impact on color outcomes. The target CIE L*, a*, and b* color values were L* = 68.5, a* = 1.43, and b* = 16.69. Using Design-Expert^®^ Software (Version 8, Stat-Ease Inc., USA), statistical analysis developed an equation to predict these values and optimize the process parameters. The ANOVA results guided a multiple linear regression analysis, incorporating parameter interactions that significantly affected predicted color outcomes in a 3-level factorial design, as detailed in Equations (2)–(4).

(2) $$L^{*}=111.17-0.026\cdot Temp-0.063\cdot Speed-1.153\cdot FeedRate+1.68E^{-004}\cdot Temp\;\cdot Speed+2.40784E^{-003}\cdot Temp\cdot FeedRate+6.98387E^{-004}\cdot Speed\;\cdot FeedRate-3.28740E^{-004}\cdot Temp^{2}$$

(3) $$a^{*}=16.18499-0.018688\cdot Speed-0.47864\cdot Feed\;rate+6.06028E^{-004}\cdot Speed\cdot FeedRate$$

(4) $$b^{*}=+19.76757-4.79457E^{-003}\cdot Speed-0.030560\cdot FeedRate$$

As shown in the above polynomial equations, which illustrated the quantitative impact of processing variables and their relations to responses, the coefficients designate the magnitude of each variable’s influence, with relations and quadratic terms embodied by multiple and higher-order coefficients correspondingly. The negative coefficients designate adverse sound effects, while hindrances and positive coefficients signify beneficial effects for optimization.

#### 3.1.3. Half-Normal Plot Evaluation Effect 

**Figure 3:**
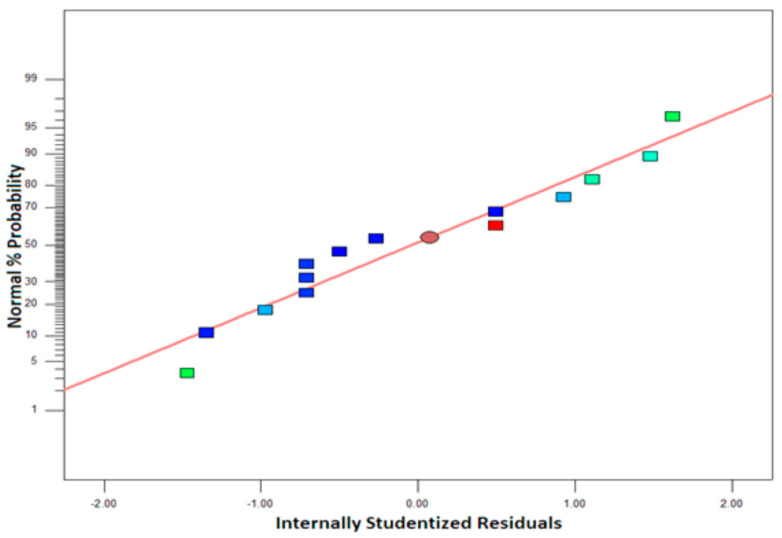
Analysis result of half-normal model.

As data points align along a straight line (Figure 3), the model depicted conforms to a normal distribution. For the result to be deemed significant, it would need to extend beyond the chart underneath the red line and to the right, suggesting that it deviates from the normal distribution. Consequently, since no effects exhibit such characteristics, no notable effects are observed in this analysis.

The variation in color in extruded polycarbonate compounds is largely affected by pigment dispersion, with poor dispersion causing visible agglomeration, leading to significant color differences, such as poor gloss, low chroma, color shifts, and poor opacity. Temperature, screw speed, feed rate, and other processing variables are important for enhancing dispersion because they influence the size and distribution of the pigment, which in turn influence the color strength, gloss, and opacity. Smaller pigment particles enhance color strength and purity due to larger surface areas. However, larger particles reflect more light, influencing sparkle and sheen. Control of particle size is essential to maintain a consistent hue and chroma.

The effects of processing parameters, especially temperature, on color clarity are depicted by half-normal plots, where most points are closely aligned with the reference line. This indicates that the residuals follow a normal distribution and that the influence of temperature on color is stable. The proximity of points to the line indicates that variations in color due to temperature changes are systematic rather than random. This suggests that controlling temperature is critical for achieving the desired color outcomes. Minimal deviations from the line indicate consistent predictions and little noise in the data. Overall, the results imply a strong and predictable relationship between temperature and color clarity in the examined materials.

#### 3.1.4. Interaction of Temperature and Feed Rate at a Fixed Speed of 750 rpm

**Figure 4:**
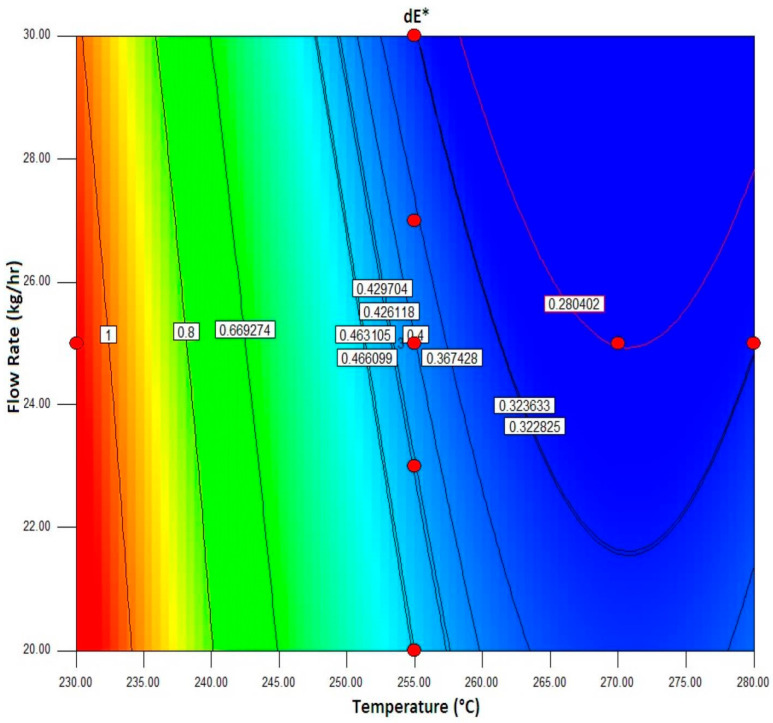
Contour plot: interaction of temperature and feed rate at a fixed speed of 750 rpm.

Figure 4 illustrates the contour plot interaction between temperature and feed rate with the speed held constant at 750 rpm. We observed that a significantly lower color value occurs when the temperature is increased while maintaining a 25 kg/h fixed feed rate. To achieve optimal pigment dispersion (reflected by the lowest dE*) at a screw speed of 750 rpm, the best combination appears to be a temperature around 260 °C and a flow rate between 24 and 28 kg/h. Deviating from these conditions—by increasing the temperature beyond 265 °C or reducing the flow rate below 22 kg/h—may result in higher dE* values and less desirable color outcomes.

Table 5 demonstrates the significance of the overall models. Temperature is the most important component of the model. Additionally, there are notable interactions between temperature and feed rate, as well as temperature and rpm. Moreover, a rise in temperature will result in a decrease in color values.

#### 3.1.5. Analysis of Variance (ANOVA) for dE*

As presented in Table 6, analysis of variance (ANOVA) exploring the minimum value of dE* was utilized to test the relations among various processing conditions and their result on the color value of dE* (L*, a*, and b*). Furthermore, statistical analysis and quadratic regression calculations were conducted using the software Design-Expert^®^ Version 8 (Stat Ease Inc., Minneapolis, Minnesota, USA).

The significance of the model is evident, as reflected by its F-value of 12.65. The probability of noise generating a “model F-value” of this magnitude is a mere 0.19%. Model terms are considered significant when their *p*-values are less than 0.05. The model’s analysis of variance (ANOVA) validated its significance against experimental data with a confidence level of 95%.

#### 3.1.6. Systematic Evaluation of Desirability

Desirability functions play a crucial role in expressing the concurrent desirability of various levels of variables, particularly in statistical and optimization contexts. Researchers and decision-makers use these functions as valuable tools to identify the ideal combination of parameters that either maximize a desired result or satisfy predetermined standards. 

**Figure 5:**
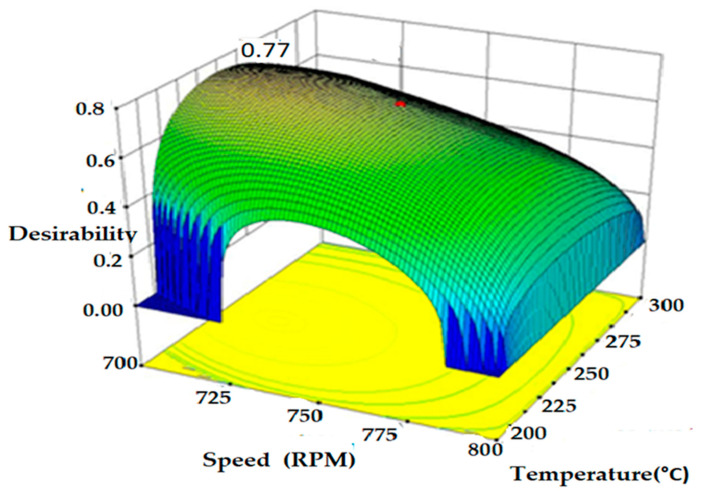
Desirability and the interactions between temperature and rpm.

In essence, desirability involves systematically evaluating and measuring the favorability of specific circumstances or results. The main goal of conducting this evaluation is to fulfill predetermined requirements or achieve specific objectives. The desirability technique is specifically employed to identify the optimal response value points. In the case of Grade 3, the determined optimal value is 0.77, as illustrated in Figure 5. The objective function in this context ranges from 0 to 1, with 1 representing the outermost point of the bounds. Numerical optimization is used to analyze the interaction between temperature and speed at a flow rate of 30 kg/h, aiming to identify a point that maximizes the desirability matrix across multiple factors and responses, utilizing Design-Expert Software (DX-8).

## 4. Rheological Characterization of Polycarbonate Composites

The compounding specimens used for the experimental test included samples tested for frequency seep and strain sweep and samples without pigment (WOP) and with pigment and additives (WP) to characterize the effects of viscosity on the output of color.

An ARES-G2 (rotational rheometer) was used to measure the viscosity, storage, and loss modulus. The dynamic viscosities’ measurements were performed in a parallel plate fixture with a diameter of 25 mm and a gap size of 1.0 mm. To ensure a linear viscoelasticity of the polymers the strain was kept at (10%). The work was conducted under a nitrogen atmosphere to prevent degradation. The melt viscosity measurements were performed between two parallel plates. Temperature was varied across at three levels: 230 °C, 255 °C, and 280 °C. All samples were compression-molded into a disk of 25 mm diameter and 1.8 mm thickness.

Initially, an ARES-G2 rheometer was used to rheologically characterize the composites in the dynamic mode under a nitrogen atmosphere at 230 °C and frequency varying from 97.43 to 0.01 Hz. Also, the initial shear rate varied from 0.5 [1/s] to 500.0 [1/s]. They were rheologically characterized using the same rheometer in three different dynamic modes: dynamic strain sweep (DSS), dynamic strain frequency sweep (DSFS), and steady sweep-rate sweep (SSRS). The temperatures tested were 230, 255, and 280 °C for a duration of 15 to 20 min until the residual stress was released and sample thermal equilibrium was attained. The test modes included strain sweep at 1% and 10 % to establish the linear strain range and dynamic frequency sweep at a strain of 10% to determine the dynamic linear viscoelastic moduli G′ G′′.

### 4.1. Dynamic Frequency Sweep–Complex Viscosity (η*)

**Figure 6:**
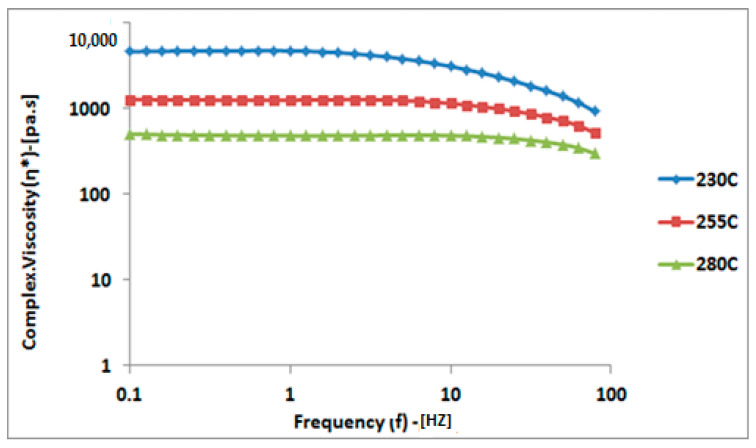
Dynamic frequency sweep—complex viscosity for PC blends at 255 °C.

Complex viscosity (η*) decreased with increasing temperature and the onset of shear thinning occurred at a higher frequency. As the frequency increased, a decrease in melt viscosity (shear thinning) was observed, as illustrated in Figure 6. 

### 4.2. Strain Sweep Test for PC Grades at 10 Hz

**Figure 7:**
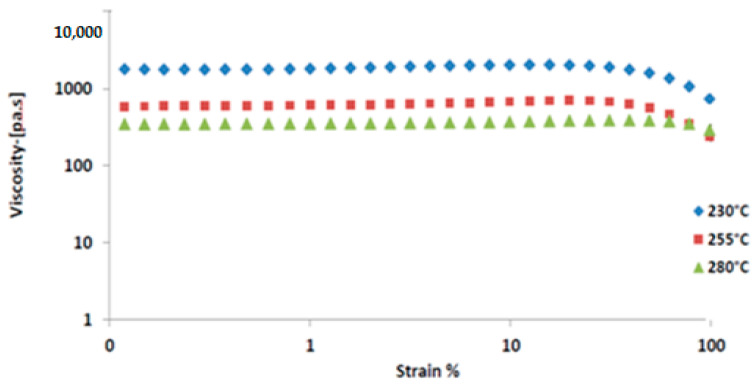
Strain sweep from the viscosity test for the PC grade at 10 Hz.

Figure 7 shows the complex viscosity as a function of strain rate, measured at a frequency of 10 Hz, with temperatures set at 230 °C, 255 °C, and 280 °C. The complex viscosity remained constant within the strain range of 0.1% to 100%, but, as expected, it decreased with increasing temperature. At lower strains, the viscosity remained stable across all temperatures, while shear thinning was observed at higher strains. The onset of shear thinning shifted to higher strain values as the temperature increased. The strain sweep test for the PC grade blends confirmed the presence of a consistent viscous region. The test results revealed that all blends exhibited linear behavior at strain magnitudes greater than 10% at various selected temperatures. The temperature increase led to a reduction in viscosity.

### 4.3. The Impact of Viscosity Behavior on Compounding—WOA and WA 

**Figure 8:**
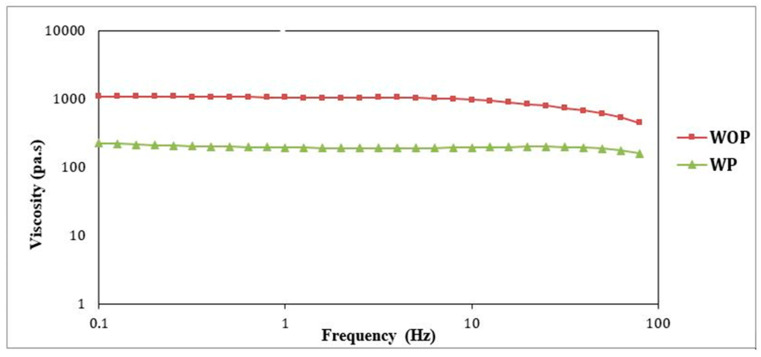
Compounds of the PC grade processed at 280 °C.

In Figure 8, we observe viscosity differences. Due to the presence of additives, WOP displayed higher viscosity, while WP showed the lowest. The variations in viscosity were influenced by processing conditions, temperature, and rheological characterization. Changes in additives and extrusion processes affected viscosity and pigment behavior, which impacted the color of the plastic on production lines. The primary factors affecting viscosity were the formulation (weight percentage) of polycarbonates and the presence of fillers, such as pigments and additives.

Figure 8 depicts viscosity curves at 280 °C for compositions ranging from 33% to 67% of PC1 and PC2 in WOP and WP blends. WOP exhibits higher viscosity, while WP shows lower viscosity, possibly due to dilution with additives. The viscosity test underscores the significant impact of temperature and fillers on the material’s rheological properties and color-matching results.

## 5. Transform Infrared Spectroscopy (FTIR) Characterization of the PC Grade

Adding a colorant not only lowers viscosity but also diminishes absorbance mechanisms, which benefits pigment dispersion. Reduced viscosity enables faster pigment wetting, leading to decreased degradation, yellowing, and a reduced color difference. The higher peak intensities for OH and CO2 in blends lacking additives and pigments (WOA) imply the presence of water or carbon, which decreases with the addition of pigments and additives (WA), resulting in enhanced transmittance and reduced haze. Correlations between FTIR and viscosity suggest that decreasing viscosity improves color matching, reducing the color difference (dE*) and enhancing color quality. The presence of water and CO2 in compounded plastic without additives impacts performance, evaluated through light transmission, haze, and color development. For WA, additives exert a more substantial influence on the peak and absorbance performance intensity of the resin grade due to the broader spectral wavelength applied.

In conclusion, FTIR microscopy stands out as a versatile technique for analyzing complex, multicomponent, and multilayer samples, particularly in heritage materials. While FTIR spectroscopy offers non-destructive stratigraphic insights, it only provides information on the presence and homogeneity of layered structures without chemical characterization of each layer. Therefore, developing FTIR microscopy-based methods for artwork analysis will offer complementary chemical details on ancient artists’ materials. In order to design experiments with consistent parameters and attain the best color results, this study makes it easier to compare FTIR characterization and rheological characteristics.

**Figure 9:**
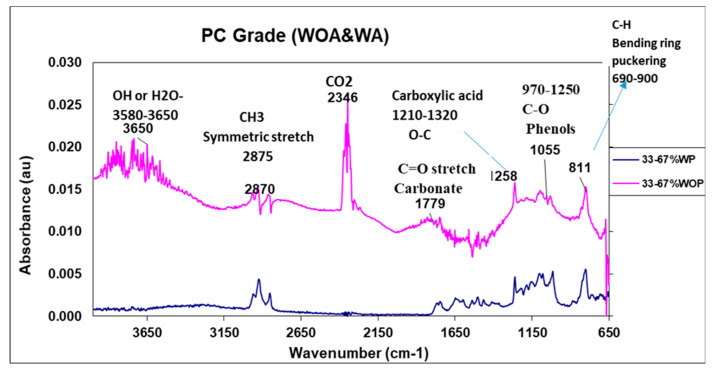
FTIR spectra for Lexan PC blend resins in two scenarios: WOA and WA.

FTIR spectra of (33/67%) Lexan PC blend resins (a) without and (b) with additives are shown in Figure 9, showing slight prominent changes in the spectral bands due to variations in PC contents. The IR curves of the polymer without additives (WOA) serve as control samples, displaying typical IR bands associated with functional groups. All spectra were normalized for peak intensity comparison. A noticeable development of OH stretch at 3580–3650 cm^−1^ and CH_3_ stretch at 2875–2870 cm^−1^ was observed. FTIR revealed the formation of carbonate molecules of CO_2_ at 2346 cm^−1^, with the CO2 linear coordination producing strong IR absorption bands in the range of 2300–2400 cm^−1^. Additionally, various carbonate species produced IR active features in the range of 1000–1800 cm^−1^, including the C=O stretch carbonate at 1670–1820 cm^−1^ and the C-O stretch (symmetric) at 1210–1320 cm^−1^. Notably, the significant development of OH stretch (3580–3650 cm^−1^) did not appear in the FTIR curve for the blends with additives and pigments (WA). 

Figure 9 displays the results of FTIR analysis of the compound containing 33 wt. % of Resin R1 (33/67%) blended with and without additives and pigments (WA and WOA). The addition of colorant (WA) resulted in decreased absorbance values compared to samples without colorant (WOA). Adding colorant could reduce both viscosity and absorbance mechanisms. Viscosity plays a significant role in pigment dispersion, with lower viscosity facilitating rapid pigment wetting, ultimately leading to decreased degradation and a reduced color difference.

## 6. Scanning Electron Microscopy (SEM) of the PC Grade at (a) 230 °C, (b) 255 °C, and (c) 280 °C

**Figure 10:**
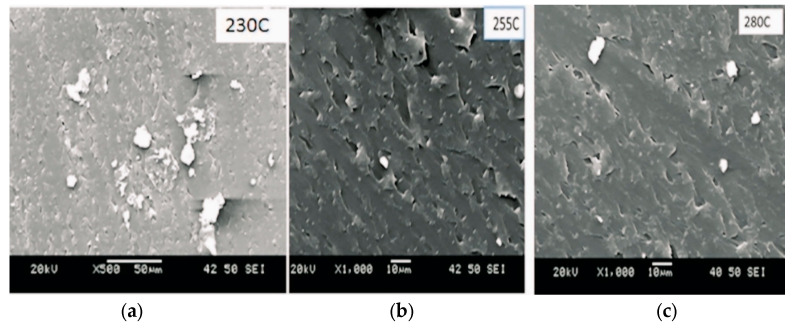
Scanning electron microscopic (SEM) images at (**a**) 230 °C: magnification is 500×, with a scale bar indicating 50 µm; (**b**) 255 °C: magnification is 1000×, with a scale bar indicating 10 µm; and (**c**) 280 °C: magnification is 1000×, with a scale bar indicating 10 µm.

As shown in Figure 10, SEM analysis was conducted to investigate the surface morphology of polycarbonate samples processed at varying temperatures. The image at 230 °C was captured at 500× magnification, with a scale bar of 50 µm, while the images at 255 °C and 280 °C were captured at 1000× magnification, with a scale bar of 10 µm. These micrographs reveal the effect of temperature on the material’s surface features and additive dispersion.

Figure 10 demonstrates consistent results, particularly emphasizing the use of elevated temperatures, with a focus on 280 °C. The aim is to achieve a steady color differentiation rate at dE* = 0.3, improve pigment wetting, reduce or prevent pigment agglomeration, and minimize color shifts. Research indicates fewer agglomerates are present at 280 °C compared to 230 °C, with higher temperatures facilitating better pigment dispersion and fewer observed agglomerates.

The viscosity of polymer melts typically decreases at elevated temperatures. This enhances their flow and mixing characteristics and improves flow, aiding in more effectively breaking up and dispersing agglomerates compared to lower temperatures, where the melt viscosity is higher. Higher temperatures also increase the solubility and diffusion rates of additives or fillers within the polymer matrix, leading to a more even distribution and fewer agglomerates. Therefore, at 280 °C, the combined effects of increased polymer chain mobility, improved viscosity and flow, enhanced solubility and diffusion rates, and potential thermal decomposition contribute to reducing agglomerates compared to lower temperatures.

## 7. Correlation of Processing Conditions and Color Output in Polycarbonate Blends

All the previous results indicate our finding of a significant relationship between processing conditions and material formulations in PC blends. This study highlights the correlation between our findings and the broader literature on PC blends, particularly regarding the impact of processing conditions on color output. Crucial elements that improve pigment dispersion and produce more consistent and vivid colors include an ideal temperature, blending speed, and feed rate. We have shown how processing temperatures have a major impact on viscosity and color matching by mixing two polycarbonate resins with different Melt Flow Indices. Using the Design of Experiments (DoE) facilitated the identification of optimal processing conditions. At the same time, techniques such as FTIR, SEM, and rheological analysis provided insights into structural features that minimize color variations and improve formulation consistency. Variations in viscosity emerged as a crucial factor influencing pigment dispersion and color consistency. The study recognized uneven colored patches due to higher viscosity that prevented the pigments from being distributed evenly, while lower viscosity facilitated better pigment dispersion under shear forces during mixing, enhancing color uniformity. The study also recognized that when the temperatures increased, lowered viscosity and improved dispersion were noted; although fluctuations could lead to color variability. Overall, our findings underscore the importance of carefully controlling processing conditions to optimize viscosity and, consequently, color output in polycarbonate blends. Our findings form a significant picture of the processing parameter–color quality relationships. Our findings are consistent with earlier research that supports customized processing techniques to attain better outcomes in polycarbonate applications.

## 8. Conclusions

This study investigates the correlation between processing conditions and color output in polycarbonate (PC) blends. Our findings highlight the significant role of temperature and shear rates in pigment dispersion. We used the Design of Experiments (DoE) technique to determine that temperature is the most critical factor affecting color quality. Higher temperatures improve pigment dispersibility and decrease viscosity. Our results show that combining different additives with PC grades improves pigment dispersion and reduces color deviations. The robustness of our model is supported by the ANOVA results, which also demonstrate how crucial processing parameters are to producing consistent color results.

Applying multiple characterization techniques, including FTIR and SEM, has deepened our understanding of material interactions that influence color quality. This study provides actionable guidelines for optimizing processing conditions, specifically recommending a temperature of approximately 260 °C; a flow rate of 24–28 kg/h; and a screw speed of 750 rpm. These insights address common issues in the plastics industry related to color mismatches, contributing to enhanced production efficiency and reduced waste.

## 9. Novel Contributions of This Study

This study identifies temperature as the most significant factor affecting color output in polycarbonate grades, demonstrating a direct correlation with viscosity and pigment dispersion. Increased shear rates lead to a reduction in viscosity, enhancing pigment dispersibility and improving color consistency in polycarbonate blends. Blending different PC grades with various additives provides a deeper understanding of how interactions among processing parameters, such as temperature and shear rates, influence viscosity and color quality.

The use of Design of Experiments (DoE) and statistical analysis (ANOVA) offers a robust framework for optimizing processing conditions, showcasing practical applications in industrial settings. Multiple characterization techniques (rheology, FTIR, and SEM) not only support the study’s findings but also enhance the understanding of material interactions that affect color output. Additionally, actionable guidelines for maintaining optimal processing conditions, including specific temperature and flow-rate ranges, are provided to achieve the best color outcomes in polycarbonate manufacturing. The findings also contribute to addressing common problems in the plastics industry related to color mismatches, potentially reducing waste and improving production efficiency.

## Figures and Tables

**Table 1:** Composition of Grade 3 (PC) material.

Formulation Types	Weight (g)	phr
Resin 1	2000	33
Resin 2	4000	67
Pigment-A (Oxide-T)	315	5.25
Pigment-B (Oxide-Zinc)	13.2	0.22
Pigment-C (Black)	2.2	0.036
Pigment-D (Red)	10.8	180
Pigment-E (Yellow)	465	7.75

**Table 2:** Percentage of adjustment of grades formulated with red pigments.

Nos.	Grade	%/Adjusted by P1	% Adjusted Caused by P1L01	No. of Adjusted Lots
1	Gr1	21.95%	-	9
2	Gr2	19.51%	-	8
3	Gr3	-	19.44%	7
4	Gr4	-	11.11%	4
5	Gr5	9.75%	-	4
6	Gr6	-	8.33%	3

**Table 3:** Parameters and experimental design levels.

Parameters	Units	3 Levels		
		Low	Medium	High
Temperature	°C	230	255	280
Speed	rpm	700	750	800
Feed rate	kg/h	20	25	30

**Table 4:** Extrusion parameters (GT) and their effects at center points (750 rpm, 25 kg/h, and 255 °C).

Extruder Run	kg/h	Temp	rpm	dE*	L*	a*	b*
1	25	255	700	0.63	68.10	1.10	15.33
2	25	255	725	0.57	68.24	1.41	15.20
3	25	255	750	0.35	68.42	1.47	15.35
4	25	255	775	0.33	68.77	1.34	15.84
5	25	255	800	0.62	68.12	1.07	15.36
6	25	230	750	1.10	67.91	1.40	14.76
7	25	240	750	0.32	68.62	1.52	15.43
8	25	255	750	0.35	68.42	1.47	15.35
9	25	270	750	0.32	68.43	1.32	15.46
10	25	280	750	0.30	68.66	1.52	15.44
11	20	255	750	0.44	68.89	1.40	15.90
12	23	255	750	0.54	68.81	1.39	15.96
13	25	255	750	0.35	68.42	1.47	15.35
14	27	255	750	0.44	68.93	1.46	15.81
15	30	255	750	0.32	68.80	1.51	15.64

**Table 5:** Interactions between temperature at a fixed feed rate and speed via color dE* (L*, a*, and b*).

Extruder-Run	kg/h	rpm	Temp.	dE*	L*	a*	b*
1	25	750	230	1.100	67.91	1.40	14.76
2	25	750	240	0.320	68.62	1.52	15.43
3	25	750	255	0.350	68.42	1.47	15.35
4	25	750	270	0.320	68.43	1.32	15.46
5	25	750	280	0.300	68.66	1.52	15.44

**Table 6:** ANOVA for the model fitted to dE*.

Source	M. S.Error (MSE)	Sum of Sources (SS) Df	*p*-Value	F-Value
Model	0.0910	0.55 6	0.0019	12.65
A (rpm)	6.59 × 10^3^	6.59 × 10^−3^ 1	0.3709	0.910
B (T)	-	- 1	0.00020	47.770
C (FR)	0.0110	00.0110 1	0.2595	1.510
(A2)	00.071	00.0710 1	0.0163	9.870
(B2)	0.140	00.140 1	0.0033	190
(C2)	6.11 × 10^5^	6.11 × 10^−5^ 1	0.9293	8.47 × 10^3^

## Data Availability

The original contributions presented in the study are included in the article, and further inquiries can be directed to the corresponding author.
